# Assessing Digital Health Implementation for a Pediatric Chronic Pain Intervention: Comparing the RE-AIM and BIT Frameworks Against Real-World Trial Data and Recommendations for Future Studies

**DOI:** 10.2196/19898

**Published:** 2020-09-01

**Authors:** Rocio de la Vega, Lee Ritterband, Tonya M Palermo

**Affiliations:** 1 Center for Child Health, Behavior and Development Seattle Children's Research Institute Seattle, WA United States; 2 Center for Behavioral Health & Technology Department of Psychiatry and Neurobehavioral Sciences University of Virginia School of Medicine Charlottesville, VA United States

**Keywords:** mobile health, eHealth, mHealth, implementation science, chronic pain, adolescents, mobile phone

## Abstract

**Background:**

Digital health interventions have demonstrated efficacy for several conditions including for pediatric chronic pain. However, the process of making interventions available to end users in an efficient and sustained way is challenging and remains a new area of research. To advance this field, comprehensive frameworks have been created.

**Objective:**

The aim of this study is to compare the Reach, Effectiveness, Adoption, Implementation, and Maintenance (RE-AIM) and Behavior Interventions using Technology (BIT) frameworks with data collected from the web-based management of adolescent pain (WebMAP Mobile; WMM) randomized controlled trial (RCT).

**Methods:**

We conducted a hybrid effectiveness-implementation cluster RCT with a stepped wedge design in which the intervention was sequentially implemented in 8 clinics, following a usual care period. Participants were 143 youths (mean age 14.5 years, SD 1.9; 117/143, 81.8% female) with chronic pain, from which 73 were randomized to receive the active intervention. Implementation outcomes were assessed using the RE-AIM and BIT frameworks.

**Results:**

According to the RE-AIM framework, the WMM showed excellent reach, recruiting a sample 19% larger than the size originally planned and consenting 79.0% (143/181) of eligible referred adolescents. Effectiveness was limited, with only global impression of change showing significantly greater improvements in the treatment group; however, greater treatment engagement was associated with greater reductions in pain and disability. Adoption was excellent (all the invited clinics participated and referred patients). Implementation was acceptable, showing good user engagement and moderate adherence and positive attitudes of providers. Costs were similar to planned, with a 7% increase in funds needed to make the WMM publicly available. Maintenance was evidenced by 56 new patients downloading the app during the maintenance period and by all clinics agreeing to continue making referrals and all, but one, making new referrals. According to the BIT, 82% (60/73) of adolescents considered the treatment acceptable. In terms of adoption, 93% (68/73) downloaded the app, and all of them used it after their first log-in. In terms of appropriateness at the user level, 2 participants were unable to download the app. Perceptions of the appearance, navigation, and theme were positive. Providers perceived the WMM as a good fit for their clinic, beneficial, helpful, and resource efficient. In terms of feasibility, no technical issues were reported. In terms of fidelity, 40% (29/73) completed the treatment. Implementation costs were 7% above the budget. With regard to penetration, 56 new users accessed the app during the maintenance period. In terms of sustainability, 88% (7/8) of clinics continued recommending the WMM after the end of the study.

**Conclusions:**

For the first time, a real-world digital health intervention was used as a proof of concept to test all the domains in the RE-AIM and BIT frameworks, allowing for comparisons.

**International Registered Report Identifier (IRRID):**

RR2-10.1016/j.cct.2018.10.003

## Introduction

### Background

Digital health interventions have demonstrated efficacy for a variety of conditions such as diabetes [1], cancer [2], chronic pain [3,4], depression, and anxiety [5]. These interventions are becoming better established and integrated within health care systems (eg, MindSpot Clinic in Australia [6]) and within clinic-based care (eg, integrating monitoring data from wearables in the digital health records) and are increasingly adopted by end users directly (eg, direct to consumer apps [7]).

However, as digital health solutions demonstrate efficacy in clinical research trials, the process of making them available to end users in an efficient way (ie, implementation in real-world settings) is still challenging and remains a new area of research. This is a key element of knowledge mobilization (ie, making evidence-based interventions available to those in need) and includes determining the best ways to design and deliver the interventions to make them easy to adapt, engaging, and low in burden for the users, while also addressing the need to secure funds for sustainability (ie, covering costs for human and technical resources).

To make advances in this field and find the most efficient ways of creating and disseminating digital health interventions, comprehensive frameworks have been created to assess both the effectiveness and implementation of these programs and to create benchmarks that allow for comparisons between different implementation and dissemination strategies. Traditional, well-established frameworks for evaluating the public health impact of interventions, such as the Reach, Effectiveness, Adoption, Implementation, and Maintenance (RE-AIM) framework [8], have been extensively used [9]. For example, *Reach* has been assessed by quantifying the number of potential clinics reached out of all potential clinics in a nationwide intervention or with the number of workers reached out of the total workers in a company; *Effectiveness* has been measured as the change in the quality of life reported by the participants of a diabetes management intervention or as the percentage of learning objectives met in a health training intervention, etc. However, a general public health impact framework may not be detailed or tailored enough to capture the unique elements and nuances of the implementation process of digital health interventions.

### Previous Work

Recently, a framework was put forth to test the implementation of “Behavior Interventions using Technology (the BIT framework, henceforth)” [10], which recharacterized implementation outcomes for behavioral intervention technology.

The BIT framework is an effort to clarify and better illustrate another widely used implementation framework, “implementation dimensions for health service interventions” [11], which includes the following domains: acceptability, adoption, appropriateness, feasibility, fidelity, implementation costs, penetration, and sustainability. The BIT framework could help fulfill the need for a more detailed and practical framework. However, although Proctor’s framework is commonly cited in digital health implementation efforts, it is not yet widely used. To our knowledge, there is only one peer-reviewed publication illustrating the different domains of the framework with examples taken from different interventions [11]. Although this is an important starting point, to date, all the domains in the BIT framework have not been tested in a single digital health trial. Further, it is unknown how the BIT framework directly compares with other established frameworks such as the RE-AIM (eg, what specific advantages may be incurred). Thus, our aim was to conduct a comprehensive proof of concept study comparing the recently developed BIT framework with the RE-AIM framework using an effectiveness-implementation trial to assess the domains, to help test their empirical validity, and to guide future research in the field.

### Aims

The primary aim of this study was to compare the RE-AIM and BIT frameworks with data collected from a hybrid effectiveness-implementation randomized controlled trial (RCT) evaluating a digital health intervention for youth with chronic pain called web-based management of adolescent pain (WebMAP Mobile, WMM; Trial Registration: ClinicalTrials.gov NCT03332563) [12]. Specifically, using this real-world trial, we aim to (1) compare and assess the strengths, limitations, and barriers of the BIT and RE-AIM frameworks and (2) make recommendations on how to design future studies to collect the data needed to test the different domains.

## Methods

### WMM Study

The WMM study is a hybrid effectiveness-implementation cluster RCT testing a mobile health intervention for self-management of adolescent chronic pain. In an effort to deliver evidence-based psychological interventions to adolescents with chronic pain, who have limited access to specialized pain clinics, a smartphone app was developed based on a well-validated web-based intervention: WebMAP; refer to the study by Palermo for details [13]). The mobile app has 6 main modules addressing the following: pain education; stress, emotions, and thoughts; relaxation and imagery; lifestyle and school interventions; staying active; and maintenance and relapse prevention and 2 supplemental modules that are assigned at baseline based on screening for sleep and mood problems. Parents can access a related web-based cognitive behavioral intervention (WebMAP parent program) that contains 8 modules, which focus on education about chronic pain, recognizing stress and negative emotions, operant strategies (2 modules), modeling, sleep hygiene and lifestyle, communication, and relapse prevention [13]. We have published the clinical trial protocol for this trial [14] and have published the main outcome paper describing the efficacy of the WMM program among youth receiving the intervention versus those receiving usual care in pain and specialty clinics [12].

#### Methods, Procedures, and Participants

For the WMM RCT, a stepped wedge design was employed [14] in which pediatric pain or specialty (eg, gastroenterology) clinics were randomized into 4 waves to have access to the intervention. A total of 8 clinics across the United States participated (5 pain clinics and 3 specialty gastroenterology clinics). Each clinic began the trial in the usual care condition, during which all youth received usual care alone. Over the subsequent 8-month period, clinics were randomly assigned to begin the intervention period (2 clinics per wave) so that all the clinics ended being exposed to the intervention, allowing a period to test for maintenance. A total of 143 youth (aged between 10 and 17 years) with chronic pain and a caregiver participated in the study, with 73 assigned to the intervention group and 70 to the usual care group.

Inclusion criteria for the main trial were purposefully broad to enhance external validity and included the following: (1) being aged between 10 and 17 years, (2) having chronic pain defined as pain present for at least 3 months, and (3) child having access to a smartphone (iOS or Android) and participating parent having access to a web-enabled device. Exclusion criteria included the following: (1) non-English speaking child or parent, (2) presently in a psychiatric crisis (eg, recent inpatient admission or suicide attempt), and (3) inability to read at the fifth-grade level per parent report. No physical or other mental health comorbidities were excluded.

#### Measures

Provider surveys, parent and child surveys, clinic data, and administrative data were collected to assess implementation outcomes. Parents reported on sociodemographic characteristics (using a background form), presence of disease-related pain, duration of pain condition, and medication history. Adolescents reported on usual pain intensity (with an 11-point numerical rating scale), pain-related disability (using the Child Activity Limitations Interview [CALI-9] [15]), and patient global impression of change (PGIC, with a single-item question: “Since the start of the study my overall status is...” [1=“No change (or condition has gotten worse)” to 7=“A great deal better, and a considerable improvement that has made all the difference.”]).

### Assessment of the Domains in the RE-AIM Framework

The RE-AIM framework has been used in different ways depending on the type of study and associated goals [9]. In this section, the metrics used to assess each of the domains and the sources used to retrieve the information for the WMM study are described.

#### Reach

Reach assesses participation in the study or intervention. It was defined as the percentage of patients giving consent to participate in the study out of the eligible patients referred by the providers at the clinics. This metric was calculated individually for each of the 8 clinics and averaged across all clinics. In addition, the planned sample size was compared with the final sample that was reached. As a goal of this study was to include a real-world population, the percentage of participants with comorbidities was tracked. Finally, acceptability of the treatment by the patients in the treatment group was assessed as a way to determine potential future reach (ie, if participants found treatment acceptable, it would be more likely to reach future patients). The Treatment Evaluation Inventory (TEI), a treatment acceptability measure used by our group in other trials [13], was administered online using Research Electronic Data Capture (REDCap) [16], a secure web-based survey app.

#### Effectiveness

Effectiveness was tested as the change from baseline to posttreatment and 3-month follow-up in patient-reported symptoms (pain and disability) using an intention-to-treat analysis and linear mixed effects (LME) regression models. Changes in PGIC were analyzed with one-way analyses of covariance. The effects of treatment engagement on treatment responses were also examined using LME regression models. The source for the effectiveness outcome measures was a battery of psychometrically sound questionnaires (described in the *Measures* section) administered online. The measures were completed independently by adolescents and their parents at the 3 time points (see WMM protocol [14] for more details).

#### Adoption

Adoption focuses on the delivery settings (ie, clinics) involved in the implementation of the intervention. It was defined as (1) the number of clinics agreeing to participate out of the clinics invited and (2) the percentage of clinics referring patients to the study out of the clinics agreeing to participate in the study.

#### Implementation

Implementation assesses the extent to which the intervention was delivered as intended. It was assessed at both the individual (ie, user and participant) and organization (ie, clinic) levels, including the cost of delivery and fidelity and consistency with how the intervention is delivered. At the *individual level*, engagement with the intervention (ie, using it as it was intended) was computed as the percentage of participants in the treatment group completing at least one module of the intervention, adherence was computed as the percentage of participants completing at least four modules, and symptom self-monitoring was also assessed as the average number of days participants registered their symptoms using the app during the treatment period. At the organization level, implementation was evaluated using a 6-item survey that assessed the attitudes of the providers of the different clinics (eg, “I think my patients would benefit from this app”) and by also assessing whether the actual costs of developing and implementing the intervention were similar to the budgeted costs or exceeded them. To test implementation at the *individual level,* app usage data, which were automatically tracked and stored, were used to determine the number of modules completed per participant. To test implementation at the organization level, a web-based survey was administered to the providers using REDCap. The projected budget was also compared with the final expense report.

#### Maintenance

Maintenance was assessed at both the individual and organization levels. The *individual level* was evaluated by assessing the symptoms at the 3-month follow-up and tracking the number of new patients starting to use the app during the maintenance period. The organization level maintenance was computed as (1) the percentage of clinics agreeing to continue referring patients to the intervention (ie, agreeing to continue to use the app in the clinic) after the study had concluded, (2) the percentage of clinics actually providing referrals of their patients to the app, and (3) the attitude of the providers’ willingness to continue prescribing the app (ie, “I will encourage my patients to use the app after the study is over”). For the *individual* and *organizational levels*, data from the web-based surveys and administrative tracking were used, respectively.

### Assessment of the Domains in the BIT Framework

In this section, the metrics chosen to assess each domain of the BIT [10] and the sources used to retrieve that information for the WMM study are described.

#### Acceptability

Acceptability is defined as the perception of the treatment as useful or satisfactory. It was assessed at both the individual and organization levels. The *individual level* was evaluated using the TEI. Specifically, we included the percentage of participants in the treatment group above the TEI cutoff total score (ie, 27) for a moderately acceptable treatment. The organization level was assessed using a provider-completed questionnaire that assessed attitudes, barriers, and facilitators to recommending the app. All information was collected using REDCap.

#### Adoption

Adoption is defined as the initiation of use of the intervention. This was measured by assessing the following: (1) the percentage of participants downloading the app on their phones, (2) the percentage of participants using the app after their first log-in, and (3) the percentage of participants completing at least one module. These data were extracted from the app server database.

#### Appropriateness

Appropriateness is the perceived relevance of fit of the intervention within a context, its compatibility with practice, and its usability. At the *individual level,* appropriateness was assessed with adolescents’ ratings on a measure of satisfaction completed posttreatment about the appearance, navigation, theme, and content of the app.

#### Feasibility

Feasibility is defined as the extent to which the intervention can be successfully used in a specific context at the individual and organization levels. At the *individual level,* we collected the number of technical issues reported during the study period. At the organization level*,* we collected the following: (1) the number of clinics agreeing to participate out of the clinics invited, (2) number of clinics referring patients out of the participating clinics, and (3) posttreatment provider feedback. Data on technical issues were retrieved from the administrative tracking system collected by the study personnel.

#### Fidelity

Fidelity is defined as the intended use versus the actual use of the intervention, or the adherence to it. We used the following: (1) the percentage of participants completing the treatment (at least four modules) and (2) the number of days tracking their symptoms in the app. Website and app back-end data were used as sources for this information.

#### Implementation Costs

Implementation costs can include any expenses related to the app or web-based program development (eg, researchers’ and developers’ salaries) or the implementation itself (eg, costs of the changes needed to adapt the intervention from a research tool to a stand-alone publicly available app). The actual costs compared with budgeted costs were computed. To assess this domain, budgets and administrative databases’ tracking expenses were used.

#### Penetration

Penetration is the integration of the practice (the intervention, in this case) within the service or clinic. To test penetration at the *individual level,* the number of adolescents using the app during the maintenance period (ie, the period after study enrollment had finished and clinicians could refer patients to the app) was calculated. To test this domain at the organization level, how many new users were referred from each clinic was calculated. This metric was retrieved from the back-end data and from the study administrative tracking data.

#### Sustainability

Finally, sustainability is defined as the extent to which the practice is maintained, its ongoing use. This was assessed in 2 different ways: (1) by calculating the number of clinics agreeing to participate in the maintenance period and (2) the percentage of clinics making referrals. This was assessed using administrative tracking data, back-end data, and budget information.

## Results

### Overview

In this section, the outcomes for the different domains of the RE-AIM and BIT frameworks are reported. Detailed results of the RE-AIM and BIT domains can be found in Tables 1 and 2, respectively.

**Table 1:** Summary of implementation outcomes using the Reach, Effectiveness, Adoption, Implementation, and Maintenance framework.

Domains		Metrices	Results	Sources
**Reach**				
	User level	Final sample out of planned sample	n=143/120 (119%)	Administrative tracking data
	User level	Consents out of eligible referred children	Total N=143/181 (79%) Clinic 1=4/4 (100%) Clinic 2=10/15 (67%) Clinic 3=4/5 (80%) Clinic 4=6/7 (86%) Clinic 5=15/17 (88%) Clinic 6=45/55 (82%) Clinic 7=15/20 (75%) Clinic 8=44/68 (76%)	Administrative tracking data
	User level	TEI^a^ mean score and percentage above 27, the moderate acceptability cutoff	Mean 30.7, 86% moderate-to-high acceptability	Patient survey
**Effectiveness**				
	User level	Change in treatment outcomes	Similar change in pain-related disability in both groups. Greater engagement associated with greater improvement in pain-related disability (Cohen *d*=-0.38 for high engagers and *d*=0.27 for low engagers). Greater improvement in global impression of change in the intervention group compared with the control group (*d*=0.54)	Patient survey
**Adoption**				
	Organization level	Percentage of invited clinics agreeing to participate	All clinics agreed (100%)	Administrative tracking data
	Organization level	Percentage of participating clinics referring patients	All clinics referred patients (100%)	Administrative tracking data
**Implementation**				
	User level	1 module (engagement)	N=54 (74%)	Back-end data
	User level	4+ modules (adherence)	N=29 (40%)	Back-end data
	User level	Number of days self-monitoring pretreatment to posttreatment	Mean 30.5 (SD 29.4); median=19; range 2-56	Back-end data
	Organization level	Provider attitudes toward the app (1 “Strongly disagree” to 5 “Strongly agree”)	Helpful to provide CBT^b^: mean 4.6 Patients would benefit: mean 4.6 Improves quality of care: mean 4.5 Better use of resources: mean 4.4 Fills an important need: mean 4.3	Provider survey
	Organization level	Actual costs compared with projected costs	The original budget was exceeded by 7%	Budget data
**Maintenance**				
	User level	New patients using the app during the maintenance period and clinic they were referred from	Total N=56 Clinic 1=7 Clinic 2=0 Clinic 3=3 Clinic 4=6 Clinic 5=26 Clinic 6=3 Clinic 7=6 Clinic 8=5	Administrative tracking data; app back-end data
	Organization level	Percentage of clinics agreeing to continue making referrals	All clinics agreed (100%)	Administrative tracking data
	Organization level	Percentage of clinics making referrals	7/8 clinics (88%) made referrals	Administrative tracking data
	Organization level	Providers: “I will encourage my patients to use the app after the study is over”	92% agreed or strongly agreed with the item	Provider survey

^a^TEI: Treatment Evaluation Inventory.

^b^CBT: cognitive behavioral therapy.

**Table 2:** Summary of implementation using the Behavior Interventions using Technology framework.

Domains		Metrices	Results	Sources
**Acceptability**				
	User level	TEI^a^ mean score and percentage above the acceptability cutoff (>27)	Mean 30.7, 86% moderate-to-high acceptability	Patient survey
	Organization level	Provider attitudes toward the app (1 “Strongly disagree” to 5 “Strongly agree”)	Helpful to provide CBT^b^: mean=4.6 My patients would benefit: mean 4.6 Improves the quality of care: mean 4.5 Better use of resources: mean 4.4 Fills in an important need: mean 4.3	Provider survey
**Adoption**				
	User level	Percentage of participants who downloaded the app	n=68/73 (93%)	Back-end data
	User level	Percentage of participants who used WMM^c^ after first log-in	n=68 (100%)	Back-end data
	User level	Percentage of participants who completed ≥1 module	n=54 (74%)	Back-end data
**Appropriateness**				
	User level	Score on app perceptions (0 “Did not like it” to 5 “Liked it very much”)	Appearance: mean 3.6 Navigation: mean 3.9 Theme: mean 3.7 Content: mean 3.3	Patient survey
**Feasibility**				
	Organization level	Percentage of clinics agreeing to continue making referrals	All clinics agreed (100%)	Administrative tracking data
	Organization level	Percentage of clinics making referrals	7/8 (88%)	Administrative tracking data
	Organization level	Final sample out of planned sample	n=143/120 (119%)	Administrative tracking data
	User level	Number of technical issues reported or complaints	n=0 (0%)	Administrative tracking data
	User level	Participants comments	Not enough space to download the app, n=2 (3%)	Patient survey
	Organization level	Providers comments	It was easy to refer patients; WMM is something useful that can be integrated in the practice	Provider survey
**Fidelity**				
	User level	Number of days tracking symptoms	Mean 30.5 (SD 29.4); median=19; range 2-56	Back-end data
	User level	Number of participants completing the treatment	n=29 (40%)	Back-end data
**Implementation costs**				
	Organization level	App development costs	As planned	Budgets
	Organization level	Making WMM publicly available	Exceed budget by 7%	Budgets
**Penetration**				
	Organization level	New patients using the app during the referral period and clinic they were referred from	Total N=56 Clinic 1=7 Clinic 2=0 Clinic 3=3 Clinic 4=6 Clinic 5=26 Clinic 6=3 Clinic 7=6 Clinic 8=5	Administrative tracking data; back-end data
**Sustainability**				
	Organization level	Referrals made	100% of the clinics agreed; 88% kept referring	Administrative tracking data
	Organization level	“I will encourage my patients to use the app after the study is over”	92% agreed or strongly agreed with the item	Provider survey

^a^TEI: Treatment Evaluation Inventory.

^b^CBT: cognitive behavioral therapy.

^c^WMM: WebMAP Mobile.

### Outcomes of the Domains in the RE-AIM Framework

#### Reach

Originally, we planned to enroll 120 adolescents and parent dyads in a 1-year period, but due to high participation rates, we were able to slightly exceed enrollment by enrolling 143 participants (ie, the final number of participants enrolled was +19% of the planned one). The percentage of consented participants out of the eligible referrals was 79% on average and ranged from 66.7% to 100% for individual clinics. The treatment was considered acceptable by 85.7% of the participants. A total of 20 adolescents (14.3%) had disease-related pain, that is, chronic pain secondary to a comorbid disease, reflecting the inclusion of a heterogeneous sample.

#### Effectiveness

Effectiveness analyses showed that pain-related disability (the main outcome) and intensity decreased in both intervention and control groups at a similar rate from baseline to posttreatment and follow-up. Adolescents in the intervention group reported significantly greater perception of improvement on the PGIC compared with youth in the control group (Cohen *d*=0.54; *P*<.001). Participants with higher engagement with the WMM (1 SD above the mean) reported significantly greater improvements in pain and pain-related disability from pretreatment to 3-month follow-up, respectively (*P*=.01 and *P*=.02; see study by Palermo et al [12] for details).

#### Adoption

All the clinics invited to participate in the study agreed to participate, and all clinics referred participants to the study (referrals ranging from 6 to 80 participants per clinic).

#### Implementation

Implementation at the *individual level* was variable. A total of 93% of participants randomized to the intervention group downloaded the app, 74% engaged with the intervention (ie, completed at least one module), and 40% were adherent to it (ie, completed at least four modules). The average number of days participants registered their symptoms was 19.7, considering that the posttreatment assessment was conducted about 8 weeks after the baseline assessment; on average, participants registered symptoms on 35.2% of days during the treatment period.

At the *organization level,* providers’ (n=27, 47% of provider sample) average scores on attitudes toward the WMM ranged from 4.3 to 4.6 out of 5, indicating strong agreement with the items related to (1) the usefulness and benefits of the WMM to patients, specifically 100% of participants agreed or strongly agreed, and (2) the cost-effectiveness of implementing the app within clinics, specifically 93% agreed or strongly agreed. The main outcomes paper includes the details [12]. Regarding budgets, the actual costs exceeded the original budget by 7% to perform a public release of the app.

#### Maintenance

At the *individual level,* symptoms at the 3-month follow-up are presented in the *Effectiveness* section. A total of 56 new patients downloaded the app during the maintenance period (with clinics referring from 0 to 26 patients each). At the organization level, 100% of the clinics agreed to continue referring patients to the intervention after the study and 88% (7/8 clinics) referred their patients to use the app during the maintenance period. Using app data tracking, we were also able to determine that in the 6-month implementation period, 56 adolescents had downloaded the app on their phones and opened it. Finally, most providers (92%) agreed or strongly agreed with the item: “I will encourage my patients to use WMM after the study is over.” Some additional comments on the survey indicated potential barriers and facilitators of implementation. The main barrier reported was that the treatment content did not seem relevant to some families. The main reported facilitators were the following: (1) it was easy to make referrals and (2) the app was perceived as something useful that they could integrate into their practice.

### Outcomes of the Domains in the BIT Framework

#### Acceptability

Treatment perceptions at the *individual level* on the TEI showed that 86% of adolescents rated the treatment as at least moderately acceptable (mean score of 31). The organization level was assessed in the same way as the *Implementation* outcome of the RE-AIM framework (ie, with the provider-completed questionnaire): all providers agreed or strongly agreed with items regarding the usefulness and benefits of the WMM to patients, and 93% of the providers agreed with the items about the cost-effectiveness of implementing the app within clinics.

#### Adoption

The percentage of participants randomized to active intervention who downloaded the app on their phones was high (68/73, 94%). All participants (100%) used the intervention after their first log-in; 74% of participants completed at least one module.

#### Appropriateness

At the *individual level*, adolescents’ responses to questions about the appearance, navigation, theme, and content of the intervention were scored 3.3 to 3.9 out of 5, on average, on a scale of 0 “Did not like it” to 5 “Liked it very much.”

#### Feasibility

Feasibility at the *individual* *level* was high: no technical issues were reported. A total of 2 adolescents (2.7%) reported not having enough space on their phones to download the app, which prevented them from using it. At the *organization level,* all clinics agreed to participate and referred participants to the study. The initial enrollment goal was exceeded by 19%. Posttreatment qualitative feedback was also positive: providers stated that it was easy to refer patients to the app and that it was a useful resource that they could integrate into their practice.

#### Fidelity

A total of 40% of the participants completed the treatment. Adolescents tracked their symptoms for 31 days on average (median 19) during the treatment, but this was highly variable (SD 29.4).

#### Implementation Costs

Implementing the app involved hiring a software development company and covering costs for code writing, graphic design, app testing, and app store fees. The original budget was US $97,500 to develop a custom app for each iOS and Android system. In addition, at the end of the study period, we decided to make the app publicly available and free of cost for the user. This last step involved some modifications (eg, eliminating the need for a password-protected log-in page and changing the privacy policy), new testing, and extending ongoing app maintenance, which exceeded our planned budget by 7% (US $12,000). This 7% was covered with donor funds and in-kind support from the app development company.

#### Penetration

Penetration resulted in 56 new users accessing the app during the maintenance period (that closed when the app was made publicly available in the stores), with the range per clinic being 0 to 26.

#### Sustainability

Finally, as a measure of sustainability, all clinics agreed to participate in the maintenance period, and we determined that 88% of them made referrals. At the end of the study, the app was made publicly available on the app stores (for Android and iOS users) of English-speaking countries free of cost for the user. Over the 2 years, the app will be maintained by the developers. As a final step for dissemination, a press release about the availability of the app was coordinated with the communication department of our institution.

### Comparison Between the Barriers and Facilitators Identified by the RE-AIM and BIT Frameworks

In Table 3, we provide a summary of our experience assessing each domain using both frameworks, including facilitators and barriers to use. We also provide recommendations on how to overcome these barriers.

**Table 3:** Lessons learned: barriers and facilitators to assess the domains of each framework and recommendations for future studies.

Framework and domains, Barriers and facilitators			Recommendations and considerations
**RE-AIM^a^**			
	**Reach**		
		Organization level (number of consents obtained out of eligible referrals received), and overall N were easy to collect because our trial involved user level referral and tracking. Availability of a standardized acceptability measure with a threshold for defining moderate acceptability facilitated this measurement.	CONSORT (The Consolidated Standards of Reporting Trials) flow diagram will provide these data. For nonresearch contexts, tracking the users approached, interested, and participating would be needed. If data from previous studies with more traditional designs or epidemiologic data are available, other metrics such as changes on comorbidities or representativeness of participants can be included. If available, the distance between participants homes and the clinic can be another way to measure Reach.
	**Effectiveness**		
		Effectiveness data from primary and secondary outcome measures was facilitated by web-based survey administration.	This domain is almost always assessed in research studies. However, it might be challenging to assess in nonresearch contexts. Low-demand approaches, such as voluntary web-based surveys could help gather information.
	**Adoption**		
		The number of centers willing to participate and participating was easily assessed with administrative tracking. We originally planned to assess the number of referrals out of the eligible participants. However, clinics were unable to provide information on the age range of their patients or how many had chronic pain; thus, the number of eligible patients is unknown.	When defining adoption, it would be key to understand availability of information required to assess this domain. If it is not, alternative metrics should be planned and collected from the beginning of the intervention.
	**Implementation**		
		Being able to access and interpret back-end data for the app required working with the developers and having a data analyst transforming the databases. Creating the web-based survey for the providers was an efficient way to collect data from multiple clinics located across the United States. Only half of providers participated in the survey, which was a barrier to understanding their perceptions.	It is important to plan the human resources needed and budget costs in advance. Web-based surveys could be a cost-effective tool to assess this metric. However, they should be brief to avoid participant burden. Qualitative feedback can be collected in face-to-face interviews or open-ended surveys but requires additional cost to analyze and interpret.
	**Maintenance**		
		We originally planned to track number of referral flyers given per clinic, but providers did not use the flyers consistently. Thus, we used alternative metrics: using app data tracking to understand number of downloads and times the app was used.	Having access to the chosen metrics should be ensured from the beginning. Ideally, objective and subjective measures (eg, asking participants if they are still using the intervention and being able to track usage with back-end data) should be collected.
**BIT^b^**			
	**Acceptability**		
		Collecting web-based acceptability feedback facilitated this assessment as it was efficient and low burden. However, we were limited to quantitative data to understand perceptions.	Web-based surveys are recommended to assess acceptability, with the same considerations that the rich detail of user perceptions may not be possible to gather in this manner.
	**Adoption**		
		Information retrieved from the app needed several steps of cleaning and restructuring databases (and the involvement of personnel with 3 different profiles and skill sets: engineers, data manager, and research scientist) before being interpretable. The costs of this process may be a barrier if unplanned for.	Working with engineers and developers from the creation of the intervention and having a dialog about the information (metrics) needed is key to ensure that adoption can be properly assessed. Budget can be a barrier because it is often expensive to obtain some metrics in a “user friendly” way (eg, the systems may provide information in a way that is difficult to understand by the lay user).
	**Appropriateness**		
		A closed-ended patient survey allowed to collect perceptions about WMM^c^ appearance, navigation, theme, and content. We were unable to capture qualitative perceptions or follow-up on the questions (they were anonymous).	For appropriateness, it would be ideal to be able to complement web-based surveys with additional qualitative assessments if costs permit their inclusion.
	**Feasibility**		
		Technical issues and complaints were carefully tracked but it is possible that additional problems were unreported.	Participants should be able to easily report technical problems to maximize the chances of reporting. A phone number or contact email (that is attended) should be provided to the participants and included in the app or website.
	**Fidelity**		
		Resources needed to use back-end data also apply for this metric.	Defining what is “intended” and “actual” use beforehand would allow decisions to be made on the metrics to use and to plan on resources if back-end data are needed to be retrieved.
	**Implementation costs**		
		We decided to compare the planned budget and the real expenses as a way to determine efficiency of the resources.	Deciding whether implementation costs were adequate can be difficult without having a reference and is study specific.
	**Penetration**		
		At the *organization level*, we planned to assess the ratio of providers and users eligible per clinic out of the total number of providers in the clinic and total users; however, the clinics were unable to provide those numbers, and we assessed this domain by calculating how many new users were referred by each clinic.	Deciding how to assess the extent to which the practice is integrated within the system can be challenging. If unknown, a pilot study could help inform what information is feasible to obtain from clinics or organizations where the intervention is being implemented.
	**Sustainability**		
		Using back-end data and checking the activation codes used, we were able to determine the percentage of clinics making referrals during the maintenance period.	Ongoing use of the intervention after the study ends can be a challenging domain to assess, because contact with the participating centers should be minimal. Collecting information in a passive way (eg, tracking use with back-end data) or with brief web-based surveys would be preferred.

^a^RE-AIM: Reach, Effectiveness, Adoption, Implementation, and Maintenance.

^b^BIT: Behavior Interventions using Technology.

^c^WMM: WebMAP Mobile.

## Discussion

### Principal Findings

This is the first time a real-world digital health intervention is used as a proof of concept to test all the domains of both the RE-AIM and BIT frameworks, demonstrating a full example of the process followed to collect information and assess the different domains from study inception to follow-up, and to compare both frameworks.

Using the RE-AIM framework, WMM [12] showed excellent *Reach*, as indicated by the high enrollment and participation rate. *Effectiveness* was limited, with only a global impression of change showing significantly greater improvements in the treatment group, although higher treatment engagement was associated with reduced pain and disability. *Adoption* was also excellent, as evidenced by the clinic participation. *Implementation* at the user level was moderate, with 93% of participants downloading the app and 40% being adherent (completing at least four modules). At the *organization level,* providers had positive attitudes toward WMM. Regarding budgets, a small increase in funds was needed to make WMM publicly available. Finally, *maintenance* at the user level was evidenced by new patients downloading the app during the maintenance period. At the *organization level,* maintenance was also excellent, with most of the clinics continuing to make new referrals.

Using the BIT framework, the *acceptability* of the WMM was evidenced by most of the users considering the treatment acceptable and the providers showing positive attitudes toward the app. *Adoption,* according to the BIT, was shown by most participants downloading the WMM and indicating its use. *Appropriateness* at the user level showed that the WMM had a good fit in this context. The *feasibility* of implementing the intervention was high, with most participants being able to download the app and not reporting any issues. At the *organization level,* the referrals exceeded the original goal; the providers mentioned that referrals were easy to make and that the WMM could be easily integrated in their practice. In addition, all clinics agreed to continue using the app with their patients, and most of them did so*. Fidelity* was moderate, given that less than half of the participants finished the treatment and only monitored symptoms on some of the days. *Implementation costs* regarding app development were as planned, but taking the extra step of making the WMM publicly available represented a small increase in costs. *Penetration* was also excellent, with new patients coming from most clinics during the implementation period. Finally, *sustainability* seems promising because most of the clinics agreed to keep using the app and did so with new patients.

Comparing the 2 frameworks, we can observe some overlap between them, but important differences exist. Specifically, RE-AIM has a strong emphasis on *Reach* and *Effectiveness* and assesses other aspects in a more limited way. In fact, an equation to calculate *Individual Impact* has been proposed [17]. It can be computed as the sum across target behaviors of the Reach domain×average of individual change at long-term follow-up (Effectiveness). Moreover, the organization level impact can be computed as Adoption×Implementation. Maintenance, however, remains as an isolated domain that is explained more superficially in the RE-AIM framework. In contrast, within the BIT framework, the concept of *maintenance* is assessed by 2 domains: penetration (integration of the practice in the context) and sustainability (the extent to which that integration is maintained). The BIT framework is more detailed in the sense that it encompasses a larger number of domains that are pertinent to the technology field and allows for testing technology-focused aspects such as usability and feasibility (providing more room to integrate qualitative user feedback), and it also considers organizational metrics and costs (which may be important for intervention and budget planning). However, the BIT fails to incorporate the effectiveness dimension, which researchers need to evaluate separately, and there are no proposals on how to integrate the various domains. In general, both frameworks provide a comprehensive assessment of the implementation of the WMM study, which was estimated to be generally successful.

Although there were several different implementation components assessed by each framework, the choice to use one over the other might depend on different contexts (either because the information required is more accessible or because the information retrieved would be more relevant for specific implementation goals). It is also important to note that most often, every dimension will not be evaluated in one project, and choices will need to be made to prioritize the dimension most central to the research question. In addition to RE-AIM and BIT, there are many other frameworks and theories for the dissemination and implementation of evidence-based interventions (the RE-AIM workgroup cites 167 different theories). The advantage of using the same implementation framework is for comparison between eHealth and mobile health interventions and to develop a common language and criteria for implementation outcomes. Our experience suggests that the RE-AIM framework might be more appropriate and easier to use for research-based interventions at either planning or evaluation stages of a project and has the advantage of being well studied over several decades with many published evaluations. The BIT framework, however, is flexible to use to study the implementation of interventions with or without an effectiveness evaluation and would also be appropriate during the planning stage of a project. The primary advantage of BIT is that it evaluates the technical and cost aspects of implementation more comprehensively and has a more nuanced focus on sustainability. However, the goals of the implementation study should be the key factors to help guide the choice of the most appropriate framework.

Both frameworks provided a structure for assessing the different domains of implementation of a digital intervention by providing guidance on what to measure (numbers to track), how to measure (suggesting some standardized metrics and cutoff thresholds, using web-based tools), and when to measure (baseline and follow-up). This would be appropriate at the planning stage to consider how to budget accordingly and at the evaluation stage to identify strengths and areas of improvement after the intervention has been implemented. The main barriers emerging for both frameworks are the potential limitations in time and budget and the unavailability of certain information depending on the context of the intervention, low participation rates in surveys, and the need of having trained personnel to interpret the retrieved data.

A number of key recommendations can be made based on this proof of concept: (1) planning ahead regarding available budget and time is crucial because assessing implementation requires additional resources; (2) communication with all members of the team and participating centers (eg, hospitals and schools) from the beginning is key to ensure access to information and to budget for the implementation study; (3) using approaches that are efficient (ie, require little time or resources) such as web-based surveys should be prioritized, but complementing with more intensive techniques (such as phone or video interviews) may be needed to capture more detailed information in certain domains; (4) conducting multisite real-world trials entails conducting research in an uncontrolled environment that can be unpredictable, and when possible, use several metrics to assess each domain, that way, if one fails, back-up metrics would be available; and (5) it may be expensive or time consuming to assess all the domains in a given framework, and choosing the domains that are relevant for the study goals before starting the trial and assessing feasibility for data collection (eg, asking the participating centers beforehand about the information they have available and are willing to share and asking developers about availability of back-end data for the lay user) would maximize chances of successful data collection and help minimize costs.

### Limitations

The limitations of this study should be considered when interpreting the findings. We were not able to assess all the domains at the *individual* and *organizational levels* for both frameworks. It is possible that studies conducted in different contexts can do so and find new barriers or facilitators not included here. Despite being a multicenter geographically diverse study, participants belonged to a specific type of population such as adolescents with chronic pain; results may be different when conducting studies with adults or with digital interventions for other health problems. Finally, the demographic characteristics of our sample of participants were predominantly White, female, and had a high socioeconomic status, which, despite being representative of adolescents attending pain clinics, may not be generalizable to samples with greater sociodemographic diversity.

### Comparison With Previous Work

To date, the RE-AIM framework has been used to assess the implementation of nondigital interventions only [9], so we do not have other digital health examples for comparison. The BIT framework has been partially tested using different intervention studies to show examples of each domain [10]; consequently, all the examples represented cases of success in assessing these domains. In previous studies, there was no specific guidance to allow precision in measuring each domain or how to problem-solve any potential issues with measurement.

As mentioned before, there are many other dissemination and implementation frameworks to assess digital interventions, such as the Non-adoption, Abandonment, Scale-up, Spread, Sustainability (NASSS) framework [18]. However, the focus of this framework is very different, being more centered on macro systems and organizations as the defining environments to test, and it is not a good fit to test implementation at the user (individual) level, missing relevant implementation information.

Future studies might consider using the structure presented here (ie, testing every domain in the frameworks using a single intervention) but including different populations and organization settings to test generalizability. It is important to test implementation in other countries that have different health care systems, because new barriers or facilitators may emerge.

### Conclusions

The RE-AIM and BIT frameworks were tested with a proof of concept study, showing both to be a useful fit for assessing implementation with different strengths and weaknesses. Some recommendations for choosing a framework to assess implementation in digital health interventions were provided. Strategies to overcome the main barriers encountered when assessing the frameworks were suggested.

## Abbreviations

BIT: Behavior Interventions using Technology
LME: linear mixed effects
PGIC: patient global impression of change
RCT: randomized controlled trial
RE-AIM: Reach, Effectiveness, Adoption, Implementation, and Maintenance
REDCap: Research Electronic Data Capture
TEI: Treatment Evaluation Inventory
WebMAP: web-based management of adolescent pain
WMM: WebMAP Mobile
